# Total protein intake and subsequent risk of chronic kidney disease: the Circulatory Risk in Communities Study

**DOI:** 10.1265/ehpm.22-00247

**Published:** 2023-05-19

**Authors:** Sachimi Kubo, Hironori Imano, Isao Muraki, Akihiko Kitamura, Hiroyuki Noda, Renzhe Cui, Koutatsu Maruyama, Kazumasa Yamagishi, Mitsumasa Umesawa, Yuji Shimizu, Mina Hayama-Terada, Masahiko Kiyama, Takeo Okada, Hiroyasu Iso

**Affiliations:** 1 Department of Cardiovascular Disease Prevention, Osaka Center for Cancer and Cardiovascular Diseases Prevention, 1-6-107 Morinomiya, Joutou-ku, Osaka 536-8588, Japan; 2 Faculty of Human Sciences, Tezukayama Gakuin University, 4-2-2 Harumidai, Minami-ku, Sakai, Osaka 590-0113, Japan; 3 Department of Public Health, Kindai University Faculty of Medicine, 377-2 Ohnohigashi, Osakasayama, Osaka 589-8511, Japan; 4 Public Health, Department of Social Medicine, Osaka University Graduate School of Medicine, 2-2 Yamadaoka, Suita, Osaka 565-0871, Japan; 5 Yao City Public Health Center, 1-2-5 Shimizu-cho, Yao, Osaka 581-0006, Japan; 6 Cabinet Secretariat, 1-6-1 Nagata-cho, Chiyoda-ku, Tokyo 100-8914, Japan; 7 Department of Internal Medicine, Okanami General Hospital, 1734 Uenokuwamachi, Iga, Mie 518-0842, Japan; 8 Laboratory of Community Health and Nutrition, Special Course of Food and Health Science, Department of Bioscience, Graduate School of Agriculture, Ehime University, 3-5-7 Tarumi, Matsuyama, Ehime 790-8566, Japan; 9 Department of Public Health Medicine, Faculty of Medicine, and Health Services Research and Development Center, University of Tsukuba, 1-1-1 Tennodai, Tsukuba, Ibaraki 305-8577, Japan; 10 Dokkyo Medical University School of Medicine, 880 Kita-kobayashi, Mibu, Shimotsugagun, Tochigi 321-0293, Japan; 11 Institute for Global Health Policy Research, Bureau of International Health Cooperation, National Center for Global Health and Medicine, 1-21-1 Toyama, Shinjuku-ku, Tokyo 162-8655, Japan

**Keywords:** Dietary protein intake, Chronic kidney disease, Japanese general population, Cohort study

## Abstract

**Background:**

Whether dietary protein intake worsens renal function in the general population has been discussed but not yet determined. We aimed to examine the longitudinal association between dietary protein intake and risk of incident chronic kidney disease (CKD).

**Methods:**

We conducted a 12-year follow-up study with 3,277 Japanese adults (1,150 men and 2,127 women) aged 40–74 years, initially free from CKD, who participated in cardiovascular risk surveys from two Japanese communities under the Circulatory Risk in Communities Study. The development of CKD was defined by the estimated glomerular filtration rate (eGFR) during the follow-up period. Protein intake was measured at baseline by using the brief-type self-administered diet history questionnaire. We estimated sex-, age-, community- and multivariate-adjusted hazard ratios (HR) for incident CKD were calculated using the Cox proportional hazards regression models according to quartiles of percentage of energy (%energy) from protein intake.

**Results:**

During 26,422 person-years of follow-up, 300 participants developed CKD (137 men and 163 women). The sex-, age-, and community-adjusted HR (95% confidence interval, CI) for the highest (≥16.9%energy) versus lowest (≤13.4%energy) quartiles of total protein intake was 0.66 (0.48–0.90), p for trend = 0.007. The multivariable HR (95%CI) was 0.72 (0.52–0.99), p for trend = 0.016 after further adjustment for body mass index, smoking status, alcohol drinking status, diastolic blood pressure, antihypertensive medication use, diabetes mellitus, serum total cholesterol levels, cholesterol-lowering medication use, total energy intake, and baseline eGFR. The association did not vary by sex, age, and baseline eGFR. When examining animal and vegetable protein intake separately, the respective multivariable HRs (95%CIs) were 0.77 (0.56–1.08), p for trend = 0.036, and 1.24 (0.89–1.75), p for trend = 0.270.

**Conclusions:**

Higher protein intake, more specifically animal protein intake was associated with a lower risk of CKD.

## Background

As renal function declines with age, the prevalence of chronic kidney disease (CKD) is expected to grow with continued population aging [1]. Approximately 347,000 individuals required dialysis in Japan at the end of 2020 [2]. Because dialysis treatment for chronic renal failure is a large burden on medical expenditure, preventing the development of chronic renal failure is highly warranted.

It is often discussed whether, in the general population, a high protein diet reduces renal function or not, but the association between high protein diet and CKD has been reported inconsistently among previous studies. In a cohort study of American nurses, higher total protein intake was not associated with renal function decline in women with normal renal function, but higher intake of non-dairy animal protein was associated with renal function decline in women with mild renal insufficiency [3]. A cohort study of Italian residents showed that higher total protein intake was associated with higher estimated glomerular filtration rate (eGFR) at baseline but with greater renal function decline over time [4] while a cohort study of Dutch adults reported no association between total protein intake and decline of renal function [5]. In American adults, higher total protein intake tended to be inversely associated with risk of CKD [6] while in Iranian adults, total protein intake was not associated with the risk [7]. A cross-sectional study of Japanese aged 30 and over reported that higher total protein intake was associated with higher eGFR [8].

Since evidence for the association between protein intake and renal function decline was mixed and unclear, we examined whether higher protein intake was associated with higher risk of CKD in the Japanese general population. Since risk of CKD increases with aging [9], and men generally have a higher risk of end-stage renal disease (ESRD) than women; the incidence of ESRD begins to rise ten years earlier in men than in women, the association was examined by sex, age, and baseline eGFR. We also examined the association of animal and vegetable intakes separately with risk of CKD because the findings from previous studies were inconsistent [3, 6, 7, 9]. Additionally, we performed the analysis, stratified by seafood, meat, egg, and dairy food intakes, the primary food sources of animal protein.

## Materials and methods

### Study population

**Fig. 1:**
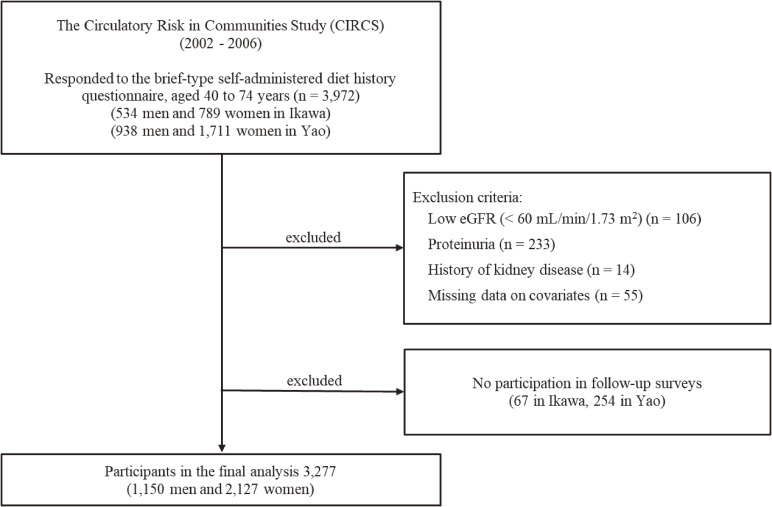
Flow chart of selection for the study participants

The Circulatory Risk in Communities Study (CIRCS) is an ongoing dynamic community cohort study involving five communities in Japan for which the study design and procedural details have been described elsewhere [10–13]. In the current study, we recruited men and women aged 40 to 74 years living in two areas of the CIRCS: the town of Ikawa (a rural community in Akita Prefecture, northwestern Japan) and the Minami-Takayasu district in Yao City (a suburb of Osaka Prefecture, midwestern Japan). As shown in Fig. 1, 3,972 individuals (534 men and 789 women from Ikawa, and 938 men and 1,711 women from Yao) responded to the brief-type self-administered diet history questionnaire (BDHQ) at the baseline survey between 2002 and 2006. We excluded the baseline participants with low eGFR (<60 mL/min/1.73 m^2^), proteinuria, or history of kidney disease, and missing data on covariates. We also excluded those who participated in no follow-up surveys. Finally, 3,277 individuals (1,150 men and 2,127 women) were enrolled in the current study and followed up until the end of 2013 to identify incident CKD.

### Nutritional survey

To assess the participants’ dietary habits during the previous month at the baseline, we used the BDHQ developed by Sasaki et al. [14], a questionnaire asking frequency of intake for 58 generally consumed foods/beverages in Japan. In the BDHQ, proteins from seafood, meat, egg, and dairy food are summed as animal protein, and proteins from grains, legumes, potatoes, confectioneries, fruits, vegetables, alcoholic and non-alcoholic beverages are summed as vegetable protein. The validity of the BDHQ for these nutrient intakes was examined; Pearson correlation coefficients for estimates of total protein intake based on single BDHQ versus the semi-weighed dietary records (DR) (four-day × four seasons) were 0.38 for men and 0.35 for women [14]. Dietary intake of total protein as well as other major nutrients were presented as percentages of energy (% energy).

### Estimation of renal function

Serum creatinine levels were assayed using an enzymatic method, and eGFR was calculated using the Chronic Kidney Disease Epidemiology Collaboration (CKD-EPI) equation with the Japanese coefficient [15, 16]. Incidence of CKD was defined as the appearance of reduced eGFR (eGFR < 60 mL/min/1.73 m^2^) during the follow-up.

### Ascertainment of other factors

Height was measured while participants were in stocking feet, and weight was determined while they wore light clothing. Body mass index (BMI) was calculated as the body weight (kg) divided by the height squared (m^2^). An interview was conducted to ascertain smoking status (never-, ex-, or current), drinking status (never-, ex-, or current drinker), histories of kidney disease, diabetes mellitus, hypertension, and hyperlipidemia, and uses of anti-diabetic, antihypertensive, and cholesterol-lowering medications. A history of kidney disease was defined as the report of kidney disorder, chronic nephritis, nephrosis, renal failure, or glomerulonephritis. Blood pressure levels were measured by trained physicians using standard mercury sphygmomanometers and standardized epidemiological methods. Serum total cholesterol and glucose were measured using enzymatic methods with an automatic analyzer (AU2700, Olympus Co., Tokyo, Japan) at the Osaka Center for Cancer and Cardiovascular Diseases Prevention, an international member of the US National Cholesterol Reference Method Laboratory Network. Diabetes mellitus was defined as a fasting glucose level of ≥7.0 mmol/L (≥126 mg/dL), a non-fasting glucose level of ≥11.1 mmol/L (≥200 mg/dL), or anti-diabetic medication use.

### Follow-up surveillance (endpoint determination)

The follow-up survey was conducted through annual cardiovascular risk surveys. For each participant, the person-years of follow-up were calculated as the duration from the date of the baseline survey through the date of incident CKD or the latest exam without incident CKD, whichever occurred first.

### Statistical analysis

According to quartiles of total protein intake, we calculated the mean values (standard deviations) and proportions of selected CKD risk factors and dietary variables at the baseline. Hazard ratios (HRs) and 95% confidence intervals (95%CIs) for incident CKD were calculated using the Cox proportional hazards regression models compared with the lowest quartile of total protein intake.

The initial model was adjusted for sex, age, and community, whereas the variables adjusted in the multivariable analysis were BMI (sex-specific quartile), smoking status (current or non-current), alcohol drinking status (current or non-current), diastolic blood pressure (continuous), antihypertensive medication use (yes or no), diabetes mellitus (yes or no), serum total cholesterol level (continuous), cholesterol-lowering medication use (yes or no), and total energy intake (sex-specific quartile) based on the findings of potential confounders from previous studies [3, 5, 6, 8, 17]. Further adjustment for baseline eGFR (60–69, 70–79, 80–89, 90 or higher) was conducted.

Additionally, we performed the analysis, stratified by sex, age (40–64 and 65–74 years old), baseline eGFR (eGFR ≥ 75 mL/min/1.73 m^2^ and 60 ≤ eGFR < 75 mL/min/1.73 m^2^), animal and vegetable protein intakes, and seafood, meat (red, processed meat, and pork), egg, and dairy food intakes as the primary food sources of animal protein.

Tests for linear trends across quartiles were conducted by modeling the median value within each quartile. The significance of the interactions by sex, age, and baseline eGFR was examined using cross-product terms of these variables with protein intake.

All statistical analyses were conducted using the SAS statistical software package, version 9.4 (SAS Institute, Inc., Cary, NC, USA). All statistical analyses were two-tailed, and a p-value < 0.05 was considered statistically significant.

## Results

During a median 8.1-year follow-up totaling 26,422 person-years, 300 CKD cases (137 for men and 163 for women; 139 in 40–64 years and 161 in 65–74 years; 191 in eGFR of 60–<75 mL/min/1.73 m^2^ and 109 in eGFR of ≥75 mL/min/1.73 m^2^) were identified.

Table 1 lists mean values and prevalence of baseline characteristics according to quartiles of total protein intake at baseline. The proportion of people in Ikawa (rural community) was higher, and that of current drinkers was lower with higher total protein intake. Mean values of animal protein, vegetable protein, and total fat intake were higher, and that of total carbohydrate intake was lower with higher total protein intake. Other characteristics did not vary according to total protein intake.

**Table 1:** Baseline CKD risk factors and selected dietary variables according to quartiles of total protein intake

	**Quartiles of total protein intake (% energy)**			

	**Quartile 1 (Low)**	**Quartile 2**	**Quartile 3**	**Quartile 4 (High)**
Total protein intake (% energy)				
Median (range)	12.2 (6.7–13.4)	14.2 (13.4–15.0)	15.8 (15.0–16.9)	18.4 (16.9–34.2)

Number of participants	819	819	820	819
Women, n (%)	544 (66.4)	523 (63.9)	533 (65.0)	527 (64.4)
Age, years, mean (SD)	58.8 (7.4)	59.0 (8.5)	58.6 (8.5)	58.9 (8.5)
Ikawa (rural community), n (%)	255 (31.1)	286 (34.9)	315 (38.4)	337 (41.2)
eGFR, mL/min per 1.73 m^2^, mean (SD)	81.3 (7.4)	81.4 (7.4)	81.7 (7.4)	81.9 (7.1)
Body mass index, kg/m^2^, mean (SD)	23.2 (3.1)	23.4 (3.1)	23.4 (3.1)	23.4 (3.1)
Current smokers, n (%)	138 (16.9)	170 (20.8)	146 (17.8)	140 (17.1)
Current drinkers, n (%)	338 (41.3)	329 (40.2)	311 (37.9)	308 (37.6)
Systolic blood pressure, mmHg, mean (SD)	128 (17)	127 (16)	126 (16)	127 (16)
Diastolic blood pressure, mmHg, mean (SD)	78 (11)	78 (11)	77 (10)	78 (10)
Use of antihypertensive medication, n (%)	143 (17.5)	154 (18.8)	144 (17.6)	142 (17.3)
Serum total cholesterol, mg/dL, mean (SD)	218 (36)	218 (35)	218 (34)	217 (37)
Use of cholesterol-lowering medication, n (%)	68 (8.3)	76 (9.3)	87 (10.6)	79 (9.7)
Diabetes mellitus, n (%)	36 (4.4)	55 (6.7)	43 (5.2)	51 (6.2)
Total energy intake, kcal/day, mean (SD)	1991 (602)	1904 (579)	1926 (535)	2026 (623)
Total protein intake, % energy, mean (SD)	12.0 (1.2)	14.2 (0.5)	15.9 (0.6)	18.9 (2.0)
Animal protein intake, % energy, mean (SD)	5.5 (1.3)	7.6 (1.0)	9.1 (1.1)	12.2 (2.3)
Vegetable protein intake, % energy, mean (SD)	6.5 (0.9)	6.6 (0.9)	6.8 (0.9)	6.7 (1.0)
Total fat intake, g/day, % energy, mean (SD)	21.3 (4.8)	25.3 (4.1)	27.4 (4.1)	29.4 (4.3)
Total carbohydrate intake, % energy, mean (SD)	58.6 (8.0)	56.1 (5.7)	53.6 (5.0)	49.1 (5.4)

CKD, chronic kidney disease; SD, standard deviation; eGFR, estimated glomerular filtration rate.

Data are presented as mean (standard deviation) or number of subjects (%).

Table 2 indicates HRs and 95% CIs of CKD according to quartiles of total protein intake. Total protein intake was inversely associated with risk of CKD in sex-, age-, and community-adjusted model; HR (95%CI) for the highest versus lowest quartiles of total protein intake was 0.66 (0.48–0.90), p for trend = 0.007. After adjustment of potential confounders including baseline eGFR, the corresponding HR (95%CI) was 0.72 (0.52–0.99), p for trend = 0.016. The inverse association was observed similarly for men and women.

**Table 2:** Hazard ratios of CKD according to quartiles of total protein intake

	**Quartiles of total protein intake (% energy)**				**P for trend**	**P for sex ** **interaction**

	**Quartile 1 ** **(Low)**	**Quartile 2**	**Quartile 3**	**Quartile 4 ** **(High)**		
Total subjects						
Number at risk	819	819	820	819		
Person years	6,322	6,396	6,738	6,966		
Number of cases	89	78	67	66		
Incidence, per 1,000 person-years	14.1	12.2	9.9	9.5		
Sex-, age-, and community-adjusted HR	1.00	0.83 (0.61–1.12)	0.70 (0.51–0.97)	0.66 (0.48–0.90)	0.007	
Multivariable HR^1^	1.00	0.82 (0.61–1.12)	0.70 (0.51–0.97)	0.66 (0.48–0.91)	0.007	
Multivariable HR^2^	1.00	0.92 (0.67–1.26)	0.66 (0.48–0.91)	0.72 (0.52–0.99)	0.016	
Men						
Number at risk	275	296	287	292		
Person years	1,902	2,170	2,238	2,438		
Number of cases	43	33	24	37		
Incidence, per 1,000 person-years	22.6	15.2	10.7	15.2		
Age-, and community-adjusted HR	1.00	0.65 (0.41–1.02)	0.47 (0.29–0.78)	0.67 (0.43–1.04)	0.064	0.850
Multivariable HR^1^	1.00	0.65 (0.41–1.04)	0.47 (0.28–0.78)	0.64 (0.41–1.00)	0.045	0.935
Multivariable HR^2^	1.00	0.79 (0.49–1.26)	0.44 (0.26–0.74)	0.71 (0.45–1.14)	0.080	0.993
Women						
Number at risk	544	523	533	527		
Person years	4,420	4,226	4,500	4,528		
Number of cases	46	45	43	29		
Incidence, per 1,000 person-years	10.4	10.6	9.6	6.4		
Age-, and community-adjusted HR	1.00	1.03 (0.68–1.55)	0.96 (0.63–1.45)	0.64 (0.40–1.02)	0.061	
Multivariable HR^1^	1.00	1.01 (0.66–1.52)	0.95 (0.62–1.46)	0.64 (0.40–1.03)	0.066	
Multivariable HR^2^	1.00	1.03 (0.67–1.58)	0.87 (0.56–1.33)	0.67 (0.42–1.08)	0.074	

CKD, chronic kidney disease; HR, hazard ratio.

In parentheses, 95% confidence intervals

Total protein intake is indicated as a percent of energy.

^1^Adjusted further for body mass index, smoking status (current or non-current), alcohol drinking status (current or non-current), diastolic blood pressure, antihypertensive medication use, diabetes mellitus, serum total cholesterol, cholesterol-lowering medication use, and total energy intake.

^2^Adjusted further for baseline estimated glomerular filtration rate.

Table 3 shows the association between total protein intake and risk of CKD, stratified by age. The inverse association was similarly observed for 40–64 years and 65–74 years, although the interaction was of borderline statistical significance in the final multivariable model.

**Table 3:** The association between total protein intake and risk of CKD, stratified by age

	**Quartiles of total protein intake (% energy)**				**P for trend**	**P for age ** **interaction**

	**Quartile 1 ** **(Low)**	**Quartile 2**	**Quartile 3**	**Quartile 4 ** **(High)**		
Ages 40–64 years						
Number at risk	571	570	595	579		
Person years	4,640	4,668	5,159	5,142		
Number of cases	41	40	27	31		
Incidence, per 1,000 person-years	8.8	8.6	5.2	6.0		
Sex-, age-, and community-adjusted HR	1.00	0.96 (0.62–1.48)	0.59 (0.36–0.96)	0.68 (0.42–1.08)	0.040	0.333
Multivariable HR^1^	1.00	0.95 (0.61–1.48)	0.58 (0.35–0.94)	0.64 (0.40–1.02)	0.022	0.424
Multivariable HR^2^	1.00	1.12 (0.71–1.76)	0.55 (0.34–0.91)	0.66 (0.40–1.09)	0.021	0.063
Ages 65–74 years						
Number at risk	248	249	225	240		
Person years	1,682	1,728	1,579	1,824		
Number of cases	48	38	40	35		
Incidence, per 1,000 person-years	28.5	22.0	25.3	19.2		
Sex-, age-, and community-adjusted HR	1.00	0.73 (0.48–1.13)	0.83 (0.54–1.26)	0.65 (0.42–1.01)	0.084	
Multivariable HR^1^	1.00	0.75 (0.49–1.15)	0.84 (0.54–1.29)	0.69 (0.44–1.07)	0.139	
Multivariable HR^2^	1.00	0.77 (0.50–1.20)	0.76 (0.49–1.20)	0.77 (0.48–1.21)	0.272	

CKD, chronic kidney disease; HR, hazard ratio.

In parentheses, 95% confidence intervals.

Total protein intake is indicated as a percent of energy.

^1^Adjusted further for body mass index, smoking status (current or non-current), alcohol drinking status (current or non-current), diastolic blood pressure, antihypertensive medication use, diabetes mellitus, serum total cholesterol, cholesterol-lowering medication use, and total energy intake.

^2^Adjusted further for baseline estimated glomerular filtration rate.

Table 4 shows the association between total protein intake and risk of CKD, stratified by baseline eGFR. The inverse association was similarly observed for baseline eGFRs of 60–<75 and ≥75 mL/min/1.73 m^2^.

**Table 4:** The association between total protein intake and risk of CKD, stratified by baseline eGFR

	**Quartiles of total protein intake (% energy)**				**P for trend**	**P for eGFR ** **interaction**

	**Quartile 1 ** **(Low)**	**Quartile 2**	**Quartile 3**	**Quartile 4 ** **(High)**		
60 ≤ eGFR < 75 mL/min/1.73 m^2^						
Number at risk	150	156	137	123		
Person years	902	955	845	781		
Number of cases	58	47	49	37		
Incidence, per 1,000 person-years	64.3	49.2	58.0	47.4		
Sex-, age-, and community-adjusted HR	1.00	0.75 (0.51–1.11)	0.91 (0.62–1.33)	0.75 (0.49–1.13)	0.262	0.636
Multivariable HR^1^	1.00	0.78 (0.52–1.16)	0.92 (0.62–1.36)	0.75 (0.49–1.14)	0.269	0.539
eGFR ≥ 75 mL/min/1.73 m^2^						
Number at risk	669	663	683	696		
Person years	5,419	5,441	5,892	6,185		
Number of cases	31	31	18	29		
Incidence, per 1,000 person-years	5.7	5.7	3.1	4.7		
Sex-, age-, and community-adjusted HR	1.00	0.93 (0.57–1.54)	0.49 (0.27–0.87)	0.73 (0.44–1.21)	0.103	
Multivariable HR^1^	1.00	0.87 (0.52–1.44)	0.47 (0.26–0.85)	0.68 (0.41–1.15)	0.071	

CKD, chronic kidney disease; HR, hazard ratio.

In parentheses, 95% confidence intervals.

Total protein intake is indicated as a percent of energy.

^1^Adjusted further for body mass index, smoking status (current or non-current), alcohol drinking status (current or non-current), diastolic blood pressure, antihypertensive medication use, diabetes mellitus, serum total cholesterol, cholesterol-lowering medication use, and total energy intake.

Table 5 indicates the associations of animal and vegetable protein intakes with risk of CKD. The inverse association with risk of CKD was confined to animal protein intake, but not vegetable protein intake.

**Table 5:** The associations of animal and vegetable protein intakes with risk of CKD

	**Quartiles of each protein intake (% energy)**				**P for trend**

	**Quartile 1 (Low)**	**Quartile 2**	**Quartile 3**	**Quartile 4 (High)**	
Animal protein intake (% energy)					
Median (range)	5.6 (0–6.7)	7.5 (6.7–8.4)	9.2 (8.4–10.3)	11.8 (10.3–29.4)	

Number at risk	819	819	820	819	
Person years	6,422	6,226	6,831	6,942	
Number of cases	82	87	61	70	
Incidence, per 1,000 person-years	12.8	14.0	8.9	10.1	
Sex-, age-, and community-adjusted HR	1.00	1.05 (0.77–1.42)	0.65 (0.47–0.91)	0.75 (0.54–1.03)	0.016
Multivariable HR^1^	1.00	1.09 (0.80–1.48)	0.67 (0.48–0.93)	0.78 (0.56–1.07)	0.026
Multivariable HR^2^	1.00	1.01 (0.74–1.37)	0.66 (0.47–0.92)	0.77 (0.56–1.08)	0.036

Vegetable protein intake (% energy)					
Median (range)	5.6 (2.9–6.0)	6.3 (6.0–6.6)	6.9 (6.6–7.2)	7.6 (7.2–12.0)	

Number at risk	819	819	820	819	
Person years	6,602	6,657	6,463	6,699	
Number of cases	68	78	77	77	
Incidence, per 1,000 person-years	10.3	11.7	11.9	11.5	
Sex-, age-, and community-adjusted HR	1.00	1.14 (0.82–1.58)	1.18 (0.85–1.63)	1.20 (0.87–1.67)	0.274
Multivariable HR^1^	1.00	1.16 (0.83–1.61)	1.17 (0.84–1.63)	1.18 (0.84–1.65)	0.360
Multivariable HR^2^	1.00	1.10 (0.79–1.53)	1.02 (0.73–1.44)	1.24 (0.89–1.75)	0.270

CKD, chronic kidney disease; HR, hazard ratio.

In parentheses, 95% confidence intervals.

Animal and vegetable protein intakes are indicated as a percent of energy.

^1^Adjusted further for body mass index, smoking status (current or non-current), alcohol drinking status (current or non-current), diastolic blood pressure, antihypertensive medication use, diabetes mellitus, serum total cholesterol, cholesterol-lowering medication use, and total energy intake.

^2^Adjusted further for baseline estimated glomerular filtration rate.

The associations of seafood, meat, egg, and dairy food intakes with risk of CKD are shown in Table 6. Seafood intake was inversely associated with risk of CKD, but meat, egg, and dairy food intakes were not associated with the risk.

**Table 6:** The associations of seafood, meat, egg, and dairy food intakes with risk of CKD

	**Quartiles of each of animal protein food source intake (g/day)**				**P for trend**

	**Quartile 1 (Low)**	**Quartile 2**	**Quartile 3**	**Quartile 4 (High)**	
Seafood intake (g/day)					
Median (range)	40.8 (0–56.1)	71.1 (56.2–87.9)	107.9 (87.9–133.7)	174.2 (133.8–609.9)	

Number at risk	819	820	819	819	
Person years	6,188	6,363	6,750	7,119	
Number of cases	94	75	66	65	
Incidence, per 1,000 person-years	15.2	11.8	9.8	9.1	
Sex-, age-, and community-adjusted HR	1.00	0.76 (0.56–1.03)	0.67 (0.49–0.92)	0.57 (0.42–0.79)	0.001
Multivariable HR^1^	1.00	0.78 (0.57–1.07)	0.65 (0.46–0.91)	0.51 (0.35–0.74)	<0.001
Multivariable HR^2^	1.00	0.77 (0.56–1.05)	0.63 (0.44–0.88)	0.50 (0.34–0.72)	<0.001

Meat intake (g/day)					
Median (range)	12.3 (0–18.0)	29.5 (18.1–35.1)	39.4 (35.1–44.8)	56.8 (44.9–327.0)	

Number at risk	819	834	803	821	
Person years	6,658	6,509	6,441	6,813	
Number of cases	82	86	57	75	
Incidence, per 1,000 person-years	12.3	13.2	8.9	11.0	
Sex-, age-, and community-adjusted HR	1.00	1.13 (0.84–1.54)	0.75 (9.54–1.06)	0.92 (0.67–1.26)	0.315
Multivariable HR^1^	1.00	1.16 (0.85–1.57)	0.77 (0.55–1.10)	0.94 (0.67–1.32)	0.045
Multivariable HR^2^	1.00	1.16 (0.85–1.59)	0.84 (0.59–1.19)	0.94 (0.66–1.32)	0.482

Egg intake (g/day)					
Median (range)	9.4 (0–18.9)	23.6 (21.2–25.9)	32.2 (26.9–52.8)	67.7 (53.7–180.5)	

Number at risk	744	835	874	824	
Person years	5,822	6,662	7,209	6,727	
Number of cases	72	66	86	76	
Incidence, per 1,000 person-years	12.4	9.9	11.9	11.3	
Sex-, age-, and community-adjusted HR	1.00	0.77 (0.55–1.07)	0.94 (0.68–1.28)	0.95 (0.69–1.31)	0.861
Multivariable HR^1^	1.00	0.78 (0.56–1.09)	0.99 (0.71–1.39)	1.03 (0.73–1.48)	0.578
Multivariable HR^2^	1.00	0.80 (0.57–1.12)	0.83 (0.59–1.15)	0.88 (0.61–1.26)	0.743

Dairy food intake (g/day)					
Median (range)	9.0 (0–47.1)	79.7 (48.2–133.5)	150.0 (134.3–171.4)	223.9 (171.4–751.9)	

Number at risk	808	823	822	824	
Person years	6,374	6,640	6,570	6,837	
Number of cases	64	79	71	86	
Incidence, per 1,000 person-years	10.0	11.9	10.8	12.6	
Sex-, age-, and community-adjusted HR	1.00	1.13 (0.81–1.57)	1.01 (0.72–1.42)	1.14 (0.83–1.58)	0.581
Multivariable HR^1^	1.00	1.10 (0.79–1.54)	1.03 (0.73–1.44)	1.20 (0.86–1.67)	0.366
Multivariable HR^2^	1.00	1.03 (0.74–1.44)	1.07 (0.76–1.52)	1.19 (0.85–1.66)	0.288

CKD, chronic kidney disease; HR, hazard ratio.

In parentheses, 95% confidence intervals.

Seafood, meat, egg and dairy food intakes are indicated as gram per day.

Meat intakes are included red, processed meat, and pork.

^1^Adjusted further for body mass index, smoking status (current or non-current), alcohol drinking status (current or non-current), diastolic blood pressure, antihypertensive medication use, diabetes mellitus, serum total cholesterol, cholesterol-lowering medication use, and total energy intake.

^2^Adjusted further for baseline estimated glomerular filtration rate.

## Discussion

This large population-based prospective study of Japanese men and women found that higher total, animal protein, and seafood intake were associated with a lower risk of incident CKD. The inverse associations did not vary by sex, age, and baseline eGFR.

Findings from previous studies regarding the association between protein intake and renal function decline were limited and inconsistent. In the Nurses’ Health Study with an 11-year follow-up of 1,624 American nurses aged 42–68 years, total protein intake was not associated with eGFR decline among women with normal renal function (eGFR ≥ 80 mL/min/1.73 m^2^), but higher intake of non-dairy animal protein was associated with greater decline of eGFR among women with mild renal insufficiency (55 ≤ eGFR < 80 mL/min/1.73 m^2^) [3]. The Gubbio Study, an Italian community-based cohort study of 1,522 men and women aged 45–64 years, showed that higher intake of total protein, estimated from 24-hour urinary urea nitrogen excretion at baseline, was associated with greater decline of eGFR [4]. The PREVEND study, a Dutch cohort study of 8,461 men and women aged 28–75 years reported no association between total protein intake estimated from 24-hour urinary urea nitrogen and eGFR decline [5]. The ARIC Study of 11,952 men and women aged 44–66 years showed that total protein intake tended to be inversely associated with risk of CKD [6], while the TLGS, an Iranian population-based prospective study of 1,630 men and women aged 27 and older, indicated no association between total protein intake and risk of CKD [7]. A cross-sectional study of 7,404 Japanese residents aged ≥30 years showed a positive association between total protein intake (either animal or vegetable protein intake) and eGFR for both men and women [8].

The associations between source-specific protein intake and risk of CKD were inconsistent. Studies from the United States and Iran showed that vegetable protein intake was inversely associated with the risk, while animal protein intake was not associated with the risk [6, 7]. The discrepancy in the findings between these studies and ours could be in part due to large differences in the distribution of animal and vegetable protein. Animal protein intake was moderately lower in Japanese than in Americans because of Japanese’s much lower meat intake and higher fish intake [18, 19]. Vegetable protein intake was much higher in Japanese than in Americans because of Japanese’s higher intake of rice as a stable food [6]. The lack of the association between vegetable protein and risk of CKD in our study may be due to a high intake and a small variation of vegetable protein. The positive association between animal protein intake and risk of CKD was observed in Americans probably because major animal protein food sources are red meat and processed meat. A 23-year follow-up of 11,952 Americans aged 44–66 years showed that red and processed meat intake was associated with an increased risk of CKD, but fish and seafood intake was associated with a reduced risk [6]. Our present study also showed that seafood intake was inversely associated with the risk of CKD, but none of meat, egg, and dairy food intakes was not associated with the risk. The protective effect of seafood intake on the development of CKD may be due to an anti-inflammatory effect of long-chain n-3 polyunsaturated fatty acids from seafood [20].

According to a 6-month randomized-clinical trial for weight reduction by modification of total protein intake under low fat intake among healthy overweight or obese men and women [21], the increased total protein intake group (n = 25, the intake changed from 91.4 to 107.8 g/day) had increased GFR (+5.2 mL/min) and increased kidney volume (+9.1 cm^3^). In contrast, the decreased total protein intake groups (n = 25, the intake changed from 91.1 to 70.4 g/day) had decreased GFR (−7.1 mL/min). A potential protective effect of total protein intake on renal function among persons without renal insufficiency may be protein-induced hyperfiltration adapted to the increased nitrogen load and the higher demand for renal clearance [22]. Our result also showed an inverse association between total protein intake and risk of CKD among persons with eGFR of 60–<75 mL/min/1.73 m^2^.

The strengths of our study included large sample size, community-based, prospective study design, repeated measurements of the outcome, and a long-term follow-up. Because of a population-based survey, our findings can be extrapolated to the Japanese general population. To the best of our knowledge, this is the first population-based prospective cohort study to examine sex- and age-specific associations of total protein intake with risk of incident CKD in Japanese.

The present study also had several limitations. First, we measured total protein intake only once at the start of this study using the BDHQ, so the time course up to the end of follow-up was unknown. Secondly, the validity of total protein intake estimated by the BDHQ was moderate, but the measurement error on the current findings was inevitable. However, the misclassification by such a measurement error is considered non-differential according to renal function, then the association between protein intake and risk of CKD may be underestimated. Thirdly, the incidence of CKD in the current study was defined as the appearance of an eGFR < 60 mL/min/1.73 m^2^. A previous study reported good repeatability for CKD diagnosis: as the coefficients of variation for serum creatinine in the present study were 0.6–0.7% [12]. However, we could not take into account 3 months or more duration of eGFR < 60 mL/min/1.73 m^2^, addressed as the clinical criteria for CKD by the Japanese Society of Nephrology [23], because no such data was available. Fourthly, at the result of the number of participation in each quartile of protein intake (% energy) during the study period, the lowest protein intake group had significantly fewer visits than the other groups (p = 0.0028). Since our present study found that the higher protein intake, the lower the risk of CKD, it could be speculated that the risk of CKD might be underestimated in the lowest protein intake group. Lastly, we had unmeasured potential confounding factors such as socioeconomic status. Higher socioeconomic status has been associated with risk of CKD [24]. According to the National Health and Nutrition Examination survey, vegetable, meat and dairy intakes were higher in people with higher household income [25].

## Conclusion

In Japanese general population, higher total protein intake, more specifically higher animal protein was associated with a lower risk of developing CKD. Our finding suggests a clinical implication for the primary prevention of CKD.

## Abbreviations

BDHQ: the brief-type self-administered diet history questionnaire
BMI: body mass index
CI: confidence interval
CIRCS: the Circulatory Risk in Communities Study
CKD: chronic kidney disease
eGFR: the estimated glomerular filtration rate
ESRD: end-stage renal disease
GFR: glomerular filtration rate
HR: hazard ratio

## Declarations

### Availability of data and material

Data cannot be shared for privacy or ethical reasons.

## References

[1] Global Burden of Disease Chronic Kidney Disease Collaboration. Global, regional, and national burden of chronic kidney disease, 1990–2017: A systematic analysis for the Global Burden of Disease Study 2017. Lancet. 2020; doi: 10.1016/S0140-6736(20)30045-3. PMC7049905 32061315

[2] Japanese Soc Dial Ther. An overview of regular dialysis treatment in Japan (as of December 31, 2020). https://docs.jsdt.or.jp/overview/file/2020/pdf/01.pdf. Published 2021. Accessed 17 Aug 2022 (in Japanese).

[3] Knight EL, Stampfer MJ, Hankinson SE, Spiegelman D, Curhan GC. The impact of protein intake on renal function decline in women with normal renal function or mild renal insufficiency. Ann Intern Med. 2003; doi: 10.7326/0003-4819-138-6-200303180-00009. 12639078

[4] Cirillo M, Lombardi C, Chiricone D, De Santo NG, Zanchetti A, Bilancio G. Protein intake and kidney function in the middle-age population: contrast between cross-sectional and longitudinal data. Nephrol Dial Transplant. 2014; doi: 10.1093/ndt/gfu056. 24658594

[5] Halbesma N, Bakker SJL, Jansen DF, Stolk RP, De Zeeuw D, De Jong PE, Gansevoort RT. High protein intake associates with cardiovascular events but not with loss of renal function. J Am Soc Nephrol. 2009; doi: 10.1681/ASN.2008060649. PMC2723984 19443643

[6] Haring B, Selvin E, Liang M, Coresh J, Grams ME, Petruski-Ivleva N, Steffen LM, Rebholz CM. Dietary protein sources and risk for incident chronic kidney disease: results from the Atherosclerosis Risk in Communities (ARIC) Study. J Ren Nutr. 2016; doi: 10.1053/j.jrn.2016.11.004. PMC5476496 28065493

[7] Alvirdizadeh S, Yuzbashian E, Mirmiran P, Eghtesadi S, Azizi F. A prospective study on total protein, plant protein and animal protein in relation to the risk of incident chronic kidney disease. BMC Nephrol. 2020; doi: 10.1186/s12882-020-02079-y. PMC7672990 33203389

[8] Higashiyama A, Watanabe M, Kokubo Y, Ono Y, Okayama A, Okamura T. Relationships between protein intake and renal function in a Japanese general population: NIPPON DATA90. J Epidemiol. 2010; doi: 10.2188/jea.JE20090222. PMC3920380 20351475

[9] Imai E, Horio M, Watanabe T, Iseki K, Yamagata K, Hara S, Ura N, Kiyohara Y, Moriyama T, Ando Y, . Prevalence of chronic kidney disease in the Japanese general population. Clin Exp Nephrol. 2009; doi: 10.1007/s10157-009-0199-x. 19513802

[10] Maruyama K, Sato S, Ohira T, Maeda K, Noda H, Kubota Y, Nishimura S, Kitamura A, Kiyama M, Okada T, . The joint impact on being overweight of self reported behaviours of eating quickly and eating until full: cross sectional survey. BMJ. 2008; doi: 10.1136/bmj.a2002. PMC2572205 18940848

[11] Imano H, Kitamura A, Sato S, Kiyama M, Ohira T, Yamagishi K, Noda H, Tanigawa T, Iso H, Shimamoto T. Trends for blood pressure and its contribution to stroke incidence in the middle-aged Japanese population: The Circulatory Risk in Communities Study (CIRCS). Stroke. 2009; doi: 10.1161/STROKEAHA.108.538629. 19342607

[12] Kubo S, Kitamura A, Imano H, Cui R, Yamagishi K, Umesawa M, Muraki I, Kiyama M, Okada T, Iso H, . Serum albumin and high-sensitivity C-reactive protein are independent risk factors of chronic kidney disease in middle-aged Japanese individuals: The circulatory risk in communities study. J Atheroscler Thromb. 2016; doi: 10.5551/jat.33530. PMC5090815 26911856

[13] Yamagishi K, Muraki I, Kubota Y, Hayama-Terada M, Imano H, Cui R, Umesawa M, Shimizu Y, Sankai T, Okada T, . The circulatory risk in communities study (CIRCS): A long-term epidemiological study for lifestyle-related disease among Japanese men and women living in communities. J Epidemiol. 2019; doi: 10.2188/jea.JE20180196. PMC6375812 30584233

[14] Kobayashi S, Honda S, Murakami K, Sasaki S, Okubo H, Hirota N, Notsu A, Fukui M, Date C. Both comprehensive and brief self-administered diet history questionnaires satisfactorily rank nutrient intakes in Japanese adults. J Epidemiol. 2012; doi: 10.2188/jea.JE20110075. PMC3798594 22343326

[15] Horio M, Imai E, Yasuda Y, Watanabe T, Matsuo S. Modification of the CKD epidemiology collaboration (CKD-EPI) equation for Japanese: accuracy and use for population estimates. Am J Kidney Dis. 2010; doi: 10.1053/j.ajkd.2010.02.344. 20416999

[16] Levey AS, Stevens LA, Schmid CH, Zhang Y, Castro AF, Feldman HI, Kusek JW, Eggers P, Van Lente F, Greene T, Coresh J. A new equation to estimate glomerular filtration rate. Ann Intern Med. 2009; doi: 10.7326/0003-4819-150-9-200905050-00006. PMC2763564 19414839

[17] Hara A, Hirata T, Okamura T, Kimura S, Urushihara H. Lifestyle behaviors associated with the initiation of renal replacement therapy disease: a retrospective cohort study using a claims database linked with specific health checkup results. Environ Health Prev Med. 2021; doi: 10.1186/s12199-021-01022-3. PMC8502396 34627137

[18] Fisheries Agency of Japan. Annual report of Fisheries Agency of Japan, March 2019 [in Japanese]. https://www.jfa.maff.go.jp/j/kikaku/wpaper/R3/attach/pdf/220603-14.pdf.

[19] Yamori Y, Sagara M, Arai Y, Kobayashi H, Kishimoto K, Matsuno I, Mori H, Mori M. Soy and fish as features of the Japanese diet and cardiovascular disease risks. PLoS One. 2017; doi: 10.1371/journal.pone.0176039. PMC5400241 28430815

[20] Gopinath B, Harris DC, Flood VM, Burlutsky G, Mitchell P. Consumption of long-chain n-3 PUFA, α-linolenic acid and fish is associated with the prevalence of chronic kidney disease. Br J Nutr. 2011; doi: 10.1017/S0007114510005040. 21255476

[21] Skov AR, Toubro S, Bülow J, Krabbe K, Parving HH, Astrup A. Changes in renal function during weight loss induced by high vs low-protein low-fat diets in overweight subjects. Int J Obes. 1999; doi: 10.1038/sj.ijo.0801048. 10578207

[22] Friedman AN. High-protein diets: Potential effects on the kidney in renal health and disease. Am J Kidney Dis. 2004; doi: 10.1053/j.ajkd.2004.08.020. 15558517

[23] Japanese Society of Nephrology. Evidence-based clinical practice guideline for CKD 2018. https://cdn.jsn.or.jp/data/CKD2018.pdf. Published 2018. Accessed 22 March 2023 (in Japanese).

[24] Vart P, Gansevoort RT, Joosten MM, Bültmann U, Reijneveld SA. Socioeconomic disparities in chronic kidney disease: a systematic review and meta-analysis. Am J Prev Med. 2015; doi: 10.1016/j.amepre.2014.11.004. 25891058

[25] Japanese Ministry of Health, Labour and Welfare. The National Health and Nutrition Survey in Japan, 2018. https://www.mhlw.go.jp/content/001066884.pdf. Published 2020. Accessed 31 Jan 2023 (in Japanese).

